# Use of Acellular Biologic Matrix Envelope for Cardiac Implantable Electronic Device Placement to Correct Migration into Submuscular Breast Implant Pocket

**DOI:** 10.1055/a-2015-8803

**Published:** 2023-03-28

**Authors:** Peyton Terry, Kenneth Bilchick, Chris A. Campbell

**Affiliations:** 1 Department of Plastic Surgery, University of Virginia, Charlottesville, Virginia; 2 Department of Internal Medicine, University of Virginia, Charlottesville, Virginia

**Keywords:** breast implant, acellular dermal matrix, cardiac device

## Abstract

Breast implants whether used for cosmetic or reconstructive purposes can be placed in pockets either above or below the pectoralis major muscle, depending on clinical circumstances such as subcutaneous tissue volume, history of radiation, and patient preference. Likewise, cardiac implantable electronic devices (CIEDs) can be placed above or below the pectoralis major muscle. When a patient has both devices, knowledge of the pocket location is important for procedural planning and for durability of device placement and performance. Here, we report a patient who previously failed subcutaneous CIED placement due to incision manipulation with prior threatened device exposure requiring plane change to subpectoral pocket. Her course was complicated by submuscular migration of the CIED into her breast implant periprosthetic pocket. With subcutaneous plane change being inadvisable due to patient noncompliance, soft tissue support of subpectoral CIED placement with an acellular biologic matrix (ABM) was performed. Similar to soft tissue support used for breast implants, submuscular CIED neo-pocket creation with ABM was performed with durable CIED device positioning confirmed at 9 months postprocedure.

## Introduction

Cardiac implantable electronic device (CIED) placement has become increasingly more prevalent over the past 30 years. 1 One relatively common complication that has increased proportionally with the use of CIEDs of subcutaneous CIED placement is migration of the CIED within or away from its original pocket site. 2 3 The risk of migration is increased with increased pocket size, tissue laxity, and weight loss, with the consequences of migration ranging from benign discomfort to uncontrolled arrhythmia or asystole. 3 Pocket revision is one technique available to restabilize CIEDs, often with novel pocket creation below the pectoralis major muscle with suture fixation. 3

Recent studies have investigated the use of acellular biologic matrix (ABM) pouches to provide better stability of CIEDs. 4 5 6 7 This technique of soft tissue support of CIEDs with biologic matrix envelopes is similar to the use of acellular dermal matrices in breast reconstruction that has been employed as an adjunct for implant-based breast reconstruction since 2005. 8 Ensuring secure placement of subpectoral CIEDs is of particular importance in patients with concomitant submuscular breast implants to avoid migration of medical devices within the periprosthetic capsule.

## Idea

This report was determined as exempt by the institution's review board for health sciences research. Patient consent was obtained postoperatively for this report and all patient identifiers were excluded for patient protection.

**Fig. 1:**
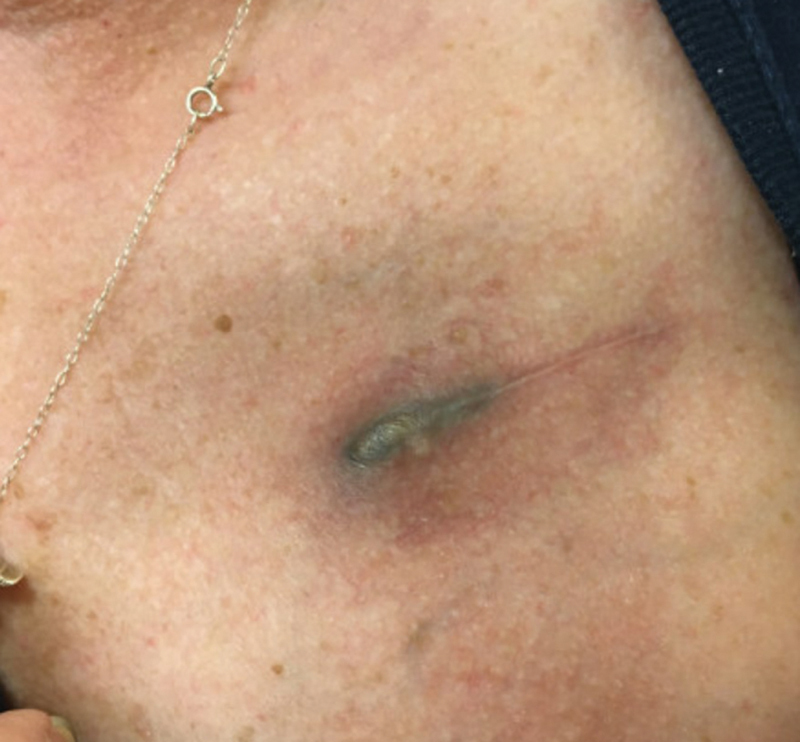
Photograph of left infraclavicular incision taken before urgent plane change. The image shows the subcutaneous cardiac implantable electronic device (CIED) with threatened exposure. Patient had endorsed frequently manipulating the incision which was thought to play a role as she had appropriate soft tissue coverage and no other risk factors for poor wound healing.

**Fig. 2:**
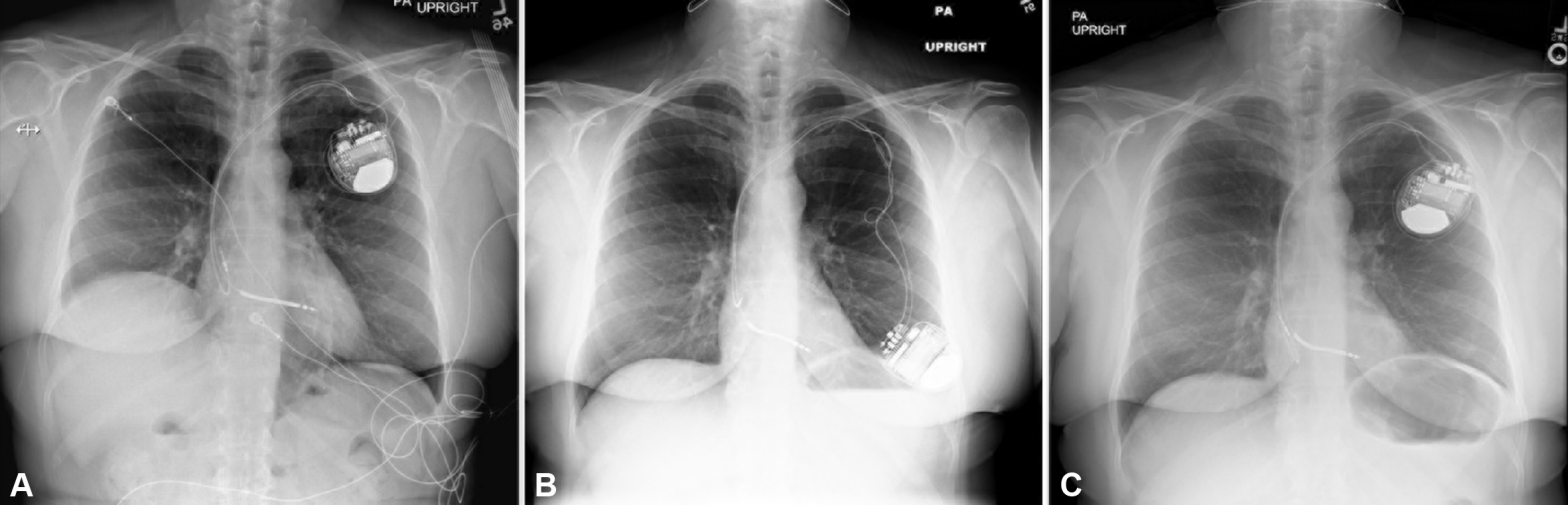
Poster-anterior (PA) X-ray images of the case patient. ( **A** ) X-ray image on the left taken 6 months before cardiac implantable electronic device (CIED) migration with CIED in appropriate position beneath pectoralis major held by suture fixation. ( **B** ) PA image in the middle panel taken at clinical presentation of CIED migration into the subpectoral breast implant pocket heralded by left chest discomfort. Note the inferior positioning of the CIED as it rests anterior to the left subpectoral breast implant. ( **C** ) PA X-ray panel on the right taken 9 months after soft tissue support of the CIED with the acellular biologic matrix envelope. Soft tissue support of the CIED with the biologic matrix envelope similar to acellular dermal matrix usage in implant-based breast reconstruction allows the CIED and breast implant to remain isolated and secured within their respective pockets.

A 57-year-old woman with a history of right-sided breast cancer underwent right-sided lumpectomy with tissue-expander and subsequent exchange for permanent implant in addition to left-sided subpectoral breast augmentation for symmetry. Over a decade later, she was diagnosed with 3rd degree atrioventricular block, and a subcutaneous left-sided dual-chamber implantable cardioverter-defibrillator (ICD) (Medtronic MRI Surescan ICD, Dual Chamber IS-1 BI, Medtronic, Minneapolis, MN) was placed. Due to persistent manipulation of the incision, there was threatened exposure of the device ( Fig. 1 ) resulting in urgent pocket revision in which the ICD was placed in a subpectoral position with suture fixation to the pectoralis minor muscle ( Fig. 2A ).

Six months later, the patient presented to the cardiology clinic with a complaint of chest discomfort. She had noticed over the last few weeks that her ICD now seemed to occupy a lower position in her left chest that was causing some pain. The patient's vital signs were stable, and she was afebrile. Physical exam revealed disruption of the normal architecture of the tissue planes concerning for an interaction between the subpectoral ICD and left breast implant without signs of infection or fluid collection.

A chest X-ray was ordered and revealed inferior displacement of the ICD suggesting that the ICD had migrated into the submuscular breast implant capsule, which occupied the same periprosthetic space ( Fig. 2B ). Informed consent was then obtained for a joint pocket revision case with cardiology and plastic surgery using an ABM envelope to secure the device as migration had occurred despite suture fixation.

The left infraclavicular incision for previous CIED pocket revision was reopened under monitored intravenous anesthesia. Subcutaneous tissue was divided with a plasma blade, and the atrioventricular leads were identified. The CIED weighed approximately 70 g and was 7 × 9 × 2 cm in dimension. The pectoralis major muscle was dissected to enter the periprosthetic cavity of the saline implant. The CIED and leads were withdrawn from the breast implant pocket and interrogated to ensure working order and replaced in an appropriate position. There was no evidence of infection.

**Fig. 3:**
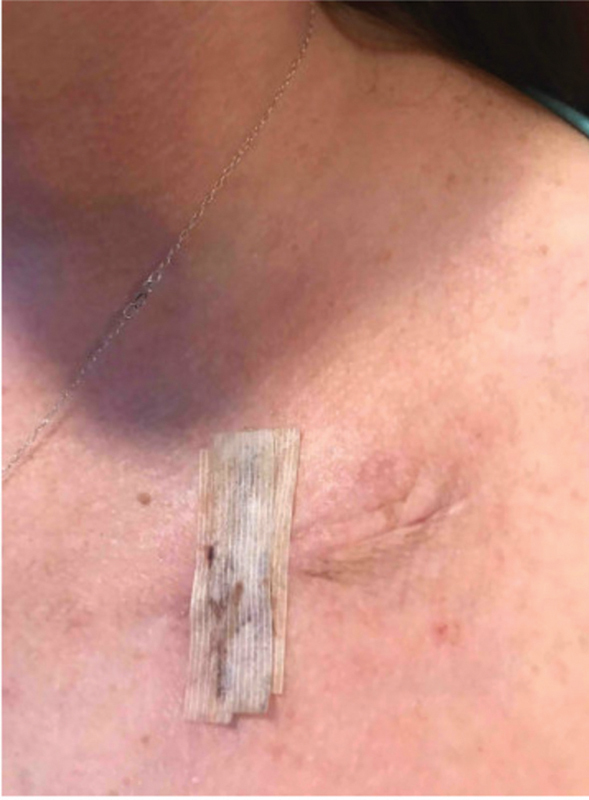
Postoperative photograph of left infraclavicular incision after acellular biologic matrix of subpectoral cardiac implantable electronic device (CIED) placement showing normal wound healing.

The pocket was then irrigated with antimicrobial solution, and the upper edge of the breast implant capsule beneath the pectoralis major was repaired with 2–0 polydioxanone (PDS) running suture. A CanGaroo Envelope (Aziyo Biologics Inc., Silver Spring, MD) bioprosthetic matrix was wrapped around the device with interrupted PDS sutures placed circumferentially around the device to provide adequate soft-tissue support. The device was secured to the pectoralis major muscle and biologic matrix with 2–0 Prolene sutures in an interrupted manner around the entire perimeter of the device. The pectoralis muscle was then closed over the device to ensure a tight, physiologically appropriate closure with 2–0 PDS suture. The remaining wound was closed with deep dermal sutures and a running subcuticular suture. The wound was dressed, and the patient was transferred to the recovery unit in stable condition. At 2-week follow-up, the wound was healing appropriately with no sign of complication ( Fig. 3 ).

Her most recent electrophysiology clinic visit and device interrogation at 9 months postprocedure showed maintained appropriate device positioning and normal CIED function with interrogation ( Fig. 2C ).

## Discussion

The use of extracellular matrices for soft tissue support of CIEDs and breast implants is a useful technique to minimize device malposition, and within cardiology is a rapidly progressing field. As demonstrated by this case, pocket positioning, appropriate soft tissue fixation, and the concomitant presence of breast implants may increase the risk or impact of subpectoral CIED displacement. These results indicate that the advantages of increased stability of bioprosthetic matrix wraps to secure CIEDs over suture fixation alone may warrant their use at primary device placement, especially in patients with the presence of a breast implant in the same submuscular pocket.

Biologic or synthetic matrix envelopes have been associated with benefits for patients with CIEDs. The recently published WRAP-IT randomized-controlled trial found decreased infection rates in CIEDs wrapped in TYRX, a synthetic tissue matrix similar to the CanGaroo Envelope, without an increased risk of complications compared with the standard of care. 6 Furthermore, while no randomized-controlled trials have been performed, in vitro and in vivo animal studies have reported decreased rates of microbial growth when using the CanGaroo Envelope, suggesting similar antimicrobial properties. 7 Animal studies have already begun to investigate the advantages and disadvantages of certain matrix materials, such as the relative rates of skin erosion in TYRX versus CanGaroo Envelope, suggesting that these materials may become specialized according to individual characteristics. 9

To date the majority of breast implant soft tissue support research has focused on acellular dermal matrices and synthetics such as poly-lactic acid and poly-4-hydroxybutyrate 8 ; however, more recent clinical research is demonstrating crossover between indications for biologic and synthetic matrices between device types. For example, a recent investigation of the use of the synthetic TYRX mesh to partially wrap breast implants showed a favorable surgical outcome profile, with the authors hypothesizing a role for mitigating the impact of capsular contracture, subclinical infection, and inflammation that may lead to breast implant-associated anaplastic large cell lymphoma. 10

As collaborative research between cardiologists and plastic surgeons continue, new indications for both synthetic and biologic materials with respect to different implantable devices are likely to increase.
